# Self-Powered Triboelectricity-Driven Multiple-Input–Single-Output Occupancy Detection System Using a Triboelectric Nanogenerator for Energy Management

**DOI:** 10.3390/polym17010034

**Published:** 2024-12-26

**Authors:** Jonghyeon Yun, Daewon Kim

**Affiliations:** 1 Department of Electronics and Information Convergence Engineering, Institute for Wearable Convergence Electronics, Kyung Hee University, 1732 Deogyeong-daero, Giheung-gu, Yongin 17104, Republic of Korea; jonghyeon.yun@khu.ac.kr; 2 Department of Electronic Engineering, Institute for Wearable Convergence Electronics, Kyung Hee University, 1732 Deogyeong-daero, Giheung-gu, Yongin 17104, Republic of Korea

**Keywords:** triboelectric nanogenerator, energy management, fiber, occupancy detection, wireless communication

## Abstract

An energy crisis, resulting from rapid population growth and advancements in the Internet of Things, has increased the importance of energy management strategies. Conventionally, energy management is conducted using sensors; however, additional energy is required to maintain sensor operation within these systems. Herein, an all-fiber-based triboelectric nanogenerator with O_2_ plasma treatment, graphene oxide/tannic acid solution coating, and hexane/1-octadecanethiol solution coating (AFT-OGH) is fabricated to implement a self-powered sensor, generating a high electrical power density, of 0.35 W/m^2^, with high stability. Using the AFT-OGH and inductors, self-powered wireless communication in real-time is implemented, achieving a communication distance of 180 cm. Based on these developments, a triboelectricity-driven multiple-input–single-output (T-MISO) system is demonstrated for the first time. An AFT-OGH-driven self-powered T-MISO occupancy detection system (AS-MODS) is implemented to determine the presence of a user in a specific space by developing a unique algorithm for automatically controlling LEDs using triboelectric signals. Considering these results, the proposed AS-MODS is expected to serve as a smart energy management system in the near future, owing to its great ability to control energy consumption.

## 1. Introduction

An energy crisis has emerged as energy demands gradually increase due to rapid population growth; furthermore, this energy crisis has been exacerbated by advancements in technologies such as the Internet of Things (IoT), because continuous energy consumption is required to maintain their operation. To address this energy crisis, possible strategies include increasing energy generation or decreasing energy consumption. Increasing energy generation is considered an attractive option because it is a simple and intuitive solution. However, conventional energy generation predominantly uses fossil fuels, contributing to global warming through carbon emissions. As an alternative, energy management is considered attractive because energy consumption is reduced through effective energy management by minimizing energy waste in systems. Energy waste occurs when lights, heating, and cooling systems continue operating even when not in use. Among these, a significant portion of energy waste occurs in the form of lighting, because lighting commonly used around us is often left on due to user carelessness, even when no one is present. The energy consumption of a single light is not significant; however, with the cumulative energy consumption of all lights in a space, such as a building, the problem increases dramatically. To eliminate this energy waste, automatic control systems have been adopted for energy management, using various sensors, such as infrared sensors, to detect occupancy and turn off lighting. Automatic control systems contribute to the reduction in wasted energy. However, additional energy is required to maintain the constant operation of sensors in these automatic control systems. In such systems, energy consumption increases proportionally with the number of sensors installed in each area, as in skyscrapers. Hence, the development of new low-power automatic control systems to detect users is required for effective energy management.

New low-power automatic control systems can be implemented by reducing the energy consumption of sensors in the systems. Energy harvester-based sensors are an attractive option for solving these issues by achieving self-powered operation through harvesting ambient energy. Recently, various energy sources, such as solar [1,2,3,4,5], heat [6,7,8,9], and mechanical movement [10,11,12,13,14,15,16,17,18], have been studied for electricity generation using energy harvesting technologies. Among them, mechanical energy harvesters are especially attractive for self-powered occupancy detection sensors, as they directly detect users by harvesting the mechanical energy generated by their movements within a space. A triboelectric nanogenerator (TENG), developed in 2012, converts mechanical energy to electrical energy through the conjugation of triboelectrification and electrostatic induction [19,20,21,22,23,24,25]. Due to their working mechanism, TENGs are fabricated using various materials such as fibers and polymers, and are capable of harvesting low-frequency mechanical energy [26,27,28,29,30,31,32]. Specifically, TENG-based sensors are attractive in IoT systems due to their self-powered property [33,34,35,36,37,38,39,40,41,42,43,44]. Hence, TENG-based self-powered systems can provide the solution to develop new low-power automatic control systems by preventing the superposition of energy consumption caused by the use of multiple sensors or modules.

Occupancy detection can be implemented in various environments, such as restrooms, offices, and homes. Therefore, wireless operation is more convenient than wired systems in terms of installation and usability. With the TENG, self-powered wireless communication systems can be implemented, enhancing the applicability of TENGs in the field of IoT platforms [34,45,46,47,48,49,50]. However, these systems possess limitations, including non-real-time wireless communication and complexity of system design. In conventional TENG-based systems, self-powered wireless communication involves a communication module such as Bluetooth, and Zigbee, an energy storage device and energy management circuit. In these systems, the energy storage device is charged by TENGs serving as energy sources to operate the communication module. To charge sufficient energy into the energy storage device, a time delay between wireless communications inevitably occurs, resulting in non-real-time wireless communication. During this time delay, important information can be lost. Hence, a self-powered, real-time wireless communication system is required to detect users with low power in real-time.

Herein, an all-fiber-based TENG (AFT) was fabricated, composed of conductive polyethylene terephthalate (PET) fabric and commercial polyester fiber. The proposed AFT was designed to possess high stability for utilization both indoors and outdoors. To achieve high stability of the AFT, a conductive PET fabric (CPF) was fabricated. Sequential surface treatments, including O_2_ plasma treatment, graphene oxide (GO)/tannic acid (TA) solution coating, and hexane/1-octadecanethiol solution coating (OGH), were conducted on the CPF (CPF-OGH). Surface properties of the CPF-OGH were investigated using scanning electron microscopy (SEM), water contact angle measurements, and sheet resistance measurements. The electrical outputs generated from the AFT using CPF-OGH (AFT-OGH) were investigated, and a high electrical power density of 0.35 W/m^2^ was generated by the AFT-OGH. Excellent stability in electrical outputs under varying frequencies and relative humidity conditions was shown by the AFT-OGH. Also, there was no significant degradation in electrical outputs generated from the AFT-OGH during a 10 h durability test. A water droplet experiment was conducted, and the AFT-OGH successfully recovered its electrical power after drying.

With these excellent properties of the AFT-OGH, facile self-powered, real-time wireless communication was implemented using inductors, capable of wireless communication up to 180 cm. Furthermore, the effects of the inductance and communication distance on signal transmission were investigated. Then, a triboelectricity-driven multiple-input–single-output (T-MISO) system for self-powered, real-time wireless communication was demonstrated for the first time, using two AFT-OGHs and two inductors. The proposed T-MISO system successfully transmitted and distinguished each signal generated by the two AFT-OGHs in real-time without any external power source. Based on these results, a unique algorithm for occupancy detection was designed, and the AFT-OGH-driven self-powered T-MISO occupancy detection system (AS-MODS) was implemented. The proposed system successfully detected user occupancy, turning the LEDs on or off based on the presence of users in the space. Considering these results, the proposed AS-MODS is expected to be utilized in the near future as a smart energy management strategy, as this system tremendously contributes to the reduction in energy consumption.

## 2. Materials and Methods

Commercial PET and polyester fabrics were utilized. For the cleaning process, a solution mixed with 5 mL of acetone, 5 mL of ethanol, and 100 mL of deionized water was used, and the bare PET fabric was dipped for 1 h and dried for 1 h at 70 °C. Meanwhile, 0.05 g of GO (Sigma-Aldrich, St. Louis, Burlington, MO, USA) and 0.125 g of TA (DUKSAN GENERAL SCIENCE, Gosanja-ro, Dongdaemun-gu, Seoul, Republic of Korea) were mixed with 100 mL of deionized water, and ultrasonication was conducted for 2 h. Before coating the GO/TA solution onto the PET fabric, O_2_ plasma treatment was conducted with a power of 100 W for 5 min. Then, the GO/TA solution was coated onto the PET fabric for 15 min and dried for 1 h at 70 °C. The CPF was fabricated after conducting Cu sputtering on this GO/TA-coated PET fabric with RF power of 100 W using Ar gas of 30 sccm. Finally, the hexane (DUKSAN GENERAL SCIENCE, Gosanja-ro, Dongdaemun-gu, Seoul, Republic of Korea) and 1-octadecanethiol (Sigma-Aldrich, St. Louis, Burlington, MO, USA) solution was coated onto the CPF-OG. By attaching the polyester fabric on the CPF, an AFT was fabricated with a size of 2 cm × 2 cm. For the AS-MODS, AFTs with a size of 5 cm × 6 cm were fabricated. To implement the T-MISO system, a load resistance of 1 GΩ and 40 mH inductors were serially connected as a transmitting part, and each transmitting part was composed of an AFT-OGH and inductor.

A scanning electron microscope (MERLIN, Carl Zeiss, Jena, Germany) was used to observe the surface of the CPF. To observe the water contact angle, Smart Drop equipment (Femtobiomed, Bundang-dong, Gyeonggi, Republic of Korea) was utilized. The electrical output was measured using an electrometer (Keithley Model 6514, Cleveland, OH, USA). To apply quantitative input force, an electrodynamic shaker (Labworks Inc., LW139.138-40, Costa Mesa, CA, USA), regulated by a signal from a function generator (Agilent Technologies Inc., 33120A, Santa Clara, CA, USA), was utilized. A linear motor with the C1100 drive (LinMot & MagSpring, Bodenäckerstrasse 2, 8957 Spreitenbach, Switzerland) was used for bending. A Python environment was utilized to implement the application.

## 3. Results

**Figure 1:**
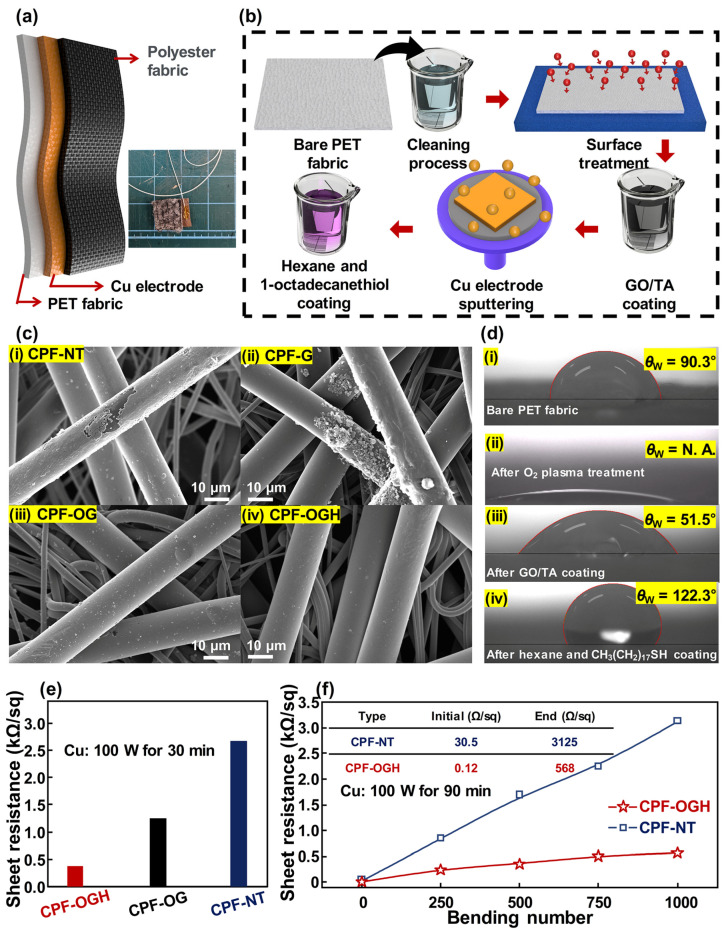
Characteristics of fabricated AFT: (**a**) Schematic of AFT. (**b**) Fabrication process of AFT. (**c**) SEM images of each CPF. (**i**) CPF-NT. (**ii**) CPF-G. (**iii**) CPF-OG. (**iv**) CPF-OGH. (**d**) Water contact angles according to sequential surface treatments on the bare PEF fabric. (**i**) Bare PET fabric. (**ii**) PET fabric after O_2_ plasma treatment. (**iii**) GO/TA-coated PET fabric. (iv) Hexane and 1-octadecanethiol-coated PET fabric. (**e**) Sheet resistances of CPF-OGH, CPF-OG, and CPF-NT. (**f**) Changes in sheet resistance of CPF-OGH and CPF-NT according to bending deformation.

Electrode stability is crucial to maintaining the electrical performance of the device, as it can easily degrade when the electrode deteriorates, even if an excellent device is fabricated. Figure 1 focuses on the demonstration of the electrical performance and stability of the fabricated electrode. Definitions of each abbreviation are provided in Table 1. An illustration of the AFT, composed of a CPF and polyester fabric, is shown in Figure 1a. The fabrication process of the AFT is described in Figure 1b. The bare PET fabric was cleaned using a mixed solution of ethanol, acetone, and deionized water. Then, O_2_ plasma treatment was conducted on the PET fabric to attach hydroxyl groups. These hydroxyl groups aid in increasing the surface energy of the PET fabric; however, they are only temporarily attached. Therefore, a GO/TA solution was coated on the PET fabric after the O_2_ plasma treatment to maintain the increased surface energy. Then, CPF was fabricated after depositing a Cu electrode through sputtering.

Since the TENG generates electricity based on the conjugation of electrostatic induction and triboelectrification, it is necessary to prevent moisture, which removes triboelectricity, to maintain the electrical performance of the TENG. Hence, a solution of hexane and 1-octadecanethiol (CH_3_(CH_2_)_17_SH) was coated on the CPF to increase its hydrophobicity. The polyester fabric was then attached to the CPF as the triboelectrification layer to fabricate the AFT. Each SEM image of the CPF is provided in Figure 1c. Due to the nano/microstructure of the PET fabric, Cu sputtering resulted in poor adhesion, causing uneven deposition of Cu on the CPF-NT surface (Figure 1c(i)). To handle this issue, the surface energy of the PET fiber was increased by coating it with a GO/TA solution. However, agglomeration of GO/TA particles was observed with the CPF-G due to the surface structure of bare PET fabric (Figure 1c(ii)). This occurs because the bare PET fabric is composed of nano/microfibers, resulting in hydrophobicity on the surface of the bare PET fabric. To solve this problem, O_2_ plasma treatment was performed to increase the surface energy of the bare PET fabric by attaching hydroxyl groups on its surface. Then, the GO/TA solution was successfully coated without any agglomeration (Figure 1c(iii)), resulting in a crack-free Cu electrode at the CPF-OG. Then, the CPF-OGH was successfully fabricated after hexane and 1-octadecanethiol coating, as shown in Figure 1c(iv). To confirm the effect of each surface treatment into the electrode deposition of the CPF, the water contact angle was investigated using a bare PET fabric, as shown in Figure 1d. The changes in surface energy of the PET fabric according to each fabrication process are provided in Figure 1d. With a water contact angle (*θ*_W_) of 90.3° (Figure 1d(i)), the bare PET fabric showed a hydrophobic surface due to its low surface energy. After conducting the O_2_ plasma treatment, the *θ*_W_ was dramatically decreased (Figure 1d(ii)) to the level where it could not be measured, indicating a significant increase in the surface energy of the PET fabric due to the attached hydroxyl groups. The *θ*_W_ of the PET fabric recovered to 51.5° after coating with the GO/TA solution, showing a hydrophilic surface (Figure 1d(iii)), indicating that coated PET fabric possesses higher surface energy than bare PET fabric. Afterward, the *θ*_W_ of the PET fabric after hexane and 1-octadecanethiol coating showed the highest value (Figure 1d(iv)) of the entire process, revealing that CPF-OGH exhibited high water resistance. As a result, the lowest sheet resistance was observed at the CPF-OGH (Figure 1e). Then, the bending test was conducted for 1000 cycles and the highest resistance against bending stress (Figure 1f) was confirmed for the CPF-OGH. Considering these results, the proposed CPF-OGH demonstrated high stability against both bending stress and humidity.

**Figure 2:**
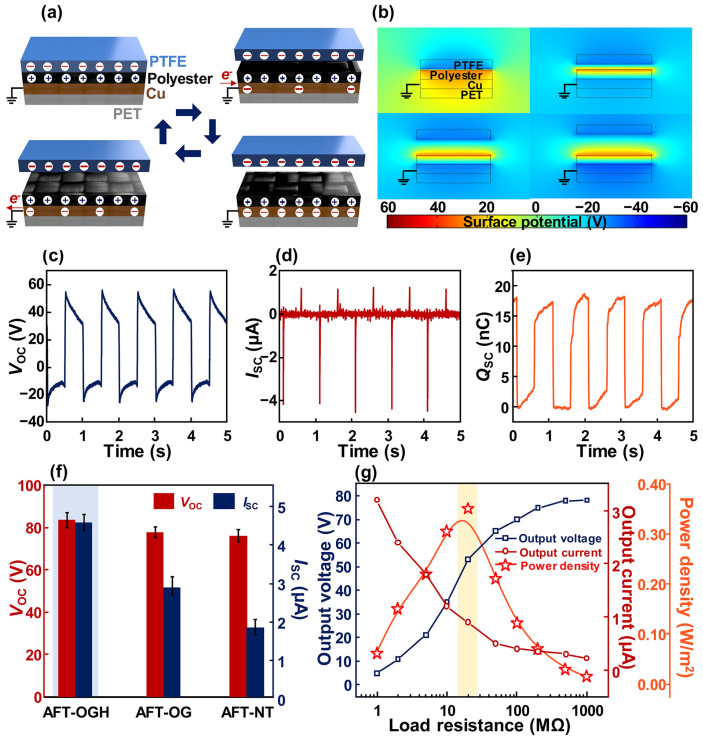
Electrical output generated from AFT-OGH: (**a**) Illustration of the working mechanism of the AFT-OGH and (**b**) its visualization using FEM simulation. (**c**) *V*_OC_, (**d**) *I*_SC_, and (**e**) *Q*sc generated from the AFT-OGH. (**f**) Comparison of the electrical outputs of AFT-OGH, AFT-OG, and AFT-NT. (**g**) Electrical power density generated from the AFT-OGH according to the load resistance.

In Figure 2, the electrical performances generated from the AFT-OGH, AFT-OG, and AFT-NT were investigated. Figure 2a illustrates the working mechanism of the proposed AFT-OGH, and its visualization is described in Figure 2b. When polytetrafluoroethylene (PTFE) contacts the polyester fabric of the AFT-OGH, the surface of the PTFE film becomes negatively charged, and positive charges appear on the polyester fabric. This occurs because PTFE contains fluorine in its structure, exhibiting high electronegativity. Due to its high electronegativity, the surface of the PTFE film tends to be negatively charged when rubbed against another material. Consequently, positive charges are induced on the polyester fabric to maintain electrical equilibrium. When the PTFE separates from the polyester fabric, the electrical equilibrium between the two materials is disrupted. To restore equilibrium, electrons are pumped from the ground to the electrode of the AFT-OGH, generating a current. When the PTFE film fully separates from the polyester film, the electrical equilibrium is re-established, and electron flow ceases. As the PTFE film approaches the polyester fabric again, the recovered electrical equilibrium begins to break, and electrons are pushed away from the electrode of the AFT-OGH to the ground, creating a current in the opposite direction during the separation process. As a result, alternating current (AC) electricity is generated from the AFT-OGH during the contact and separation process.

Based on this working mechanism, the electrical outputs generated from the AFT-OGH were investigated in Figure 2c–e, and the AFT-OGH generated an open-circuit voltage (*V*_OC_) of 83 V, short-circuit current (*I*_SC_) of 4.5 μA, and transferred charge in short-circuit state (*Q*_SC_) of 18.5 nC. In Figure 2f, a comparison of the electrical outputs generated from the AFT-OGH, AFT-OG, and AFT-NT is presented. The *V*_OC_ values generated from each AFT showed similar values. However, the *I*_SC_ values differed among the samples. This difference is due to the resistance of the CPF. The voltage is determined by the potential difference between two contact materials in the TENG. Hence, despite the differences in sheet resistances of each CPF, the occurrences of triboelectric-induced charges on the surfaces between two materials is similar, indicating that each AFT generates a similar *V*_OC_ value. In contrast, the *I*_SC_ generated from the AFT-OGH is affected by sheet resistance because high sheet resistance impedes the movement of charges. Consequently, the highest *I*_SC_ was measured with the AFT-OGH, fabricated based on the CPF-OGH, which exhibits the lowest sheet resistance among the samples. In Figure 2g, the electrical power density was investigated according to the load resistance, and a high electrical power density of 0.35 W/m^2^ was generated. Since the AFT-OGH and AFT-OG have the same electrode deposition process, the maximum power was generated at a load resistance of 20 MΩ, as shown in Table S1. On the other hand, the AFT-NT showed poor electrode adhesion and high sheet resistance. Therefore, its internal resistance is higher than that of the AFT-OGH and AFT-OG, and the load resistance generating maximum power density is increased to 50 MΩ. In terms of output current, even though the AFT-OGH and AFT-OG were fabricated with the same electrode deposition process, the AFT-OGH generated higher current and voltage than the AFT-OG due to its hydrophobic coating. This seems to be a difference that occurred due to the minimal influence of humidity. As a result, the highest power density occurred in the AFT-OGH. Based on these results, among the AFT-OGH, AFT-OG, and AFT-NT, the AFT-OGH was selected for use as the self-powered sensor.

**Figure 3:**
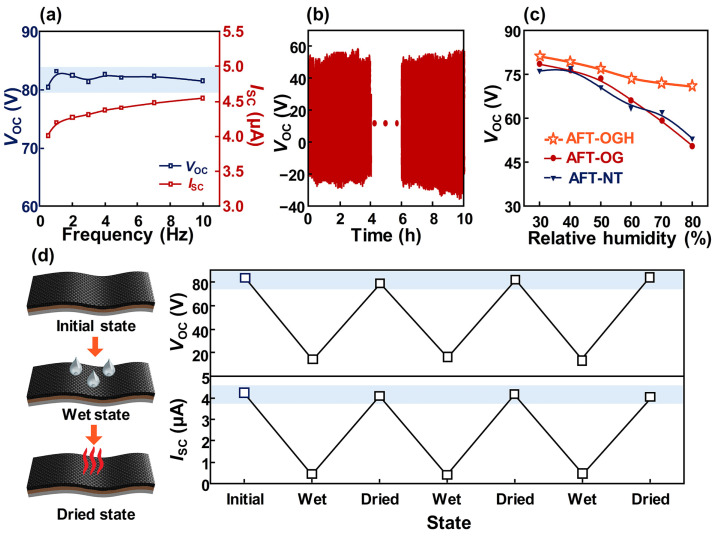
Stability in electrical output generated from the AFT-OGH: (**a**) The *V*_OC_ and *I*_SC_ generated from the AFT-OGH according to the frequency. (**b**) Durability of the AFT-OGH. (**c**) Electrical output of the AFT-OGH with various relative humidity. (**d**) Illustration of water droplet experiment and its experimental results.

Figure 3 mainly illustrates the stability of the AFT-OGH’s electrical performance. In Figure 3a, the electrical outputs generated from the AFT-OGH were investigated by varying the input frequency. The AFT-OGH generated similar *V*_OC_ values when the input frequency was changed in the range of 0.5 to 10 Hz. In contrast, the *I*_SC_ generated from the AFT-OGH increased as the input frequency increased. This differing trend between the *V*_OC_ and *I*_SC_ is due to the working mechanism of the TENG. According to previous studies, the electrical outputs generated from the TENG are quantified by the following equations [29]:

(1) $$V_{\mathrm{OC}}=\frac{\sigma x(t)}{\varepsilon_{0}}$$

(2) $$Q_{\mathrm{SC}}=\frac{S\sigma x(t)}{d_{0}+x(t)}$$

(3) $$I_{\mathrm{SC}}=\frac{dQ_{\mathrm{SC}}}{dt}$$

where *σ* is the surface charge density, *x*(*t*) is the distance between the two triboelectric layers, *d*_0_ is the thickness of the dielectric material, *ε*_0_ is the dielectric constant, and S is the contact area. Hence, while the AFT-OGH generated similar *V*_OC_ values regardless of the input frequency, the generated *I*_SC_ varied with frequency. Therefore, the voltage was selected as a signal because the AFT-OGH can generate the voltage with similar amplitude, regardless of the input frequency. A durability test was conducted with the AFT-OGH for 10 h to show its excellent stability, and no significant degradation was observed during the test (Figure 3b). Furthermore, the AFT-OGH showed the lowest change rate in voltage under humid environments among the AFT-OGH, AFT-OG, and AFT-NT, as shown in Figure 3c. This result can be explained by the effect of the hexane and 1-octadecanethiol coating onto the AFT-OGH. Fibers composing the AFT inherently retain some moisture; however, due to the high hydrophobicity property of the AFT-OGH, the voltage generated showed the lowest change rate. Then, water droplets were dropped on the AFT-OGH and dried, while the electrical output was measured at each step, repeated three times (Figure 3d). When water droplets were present on the AFT-OGH, the electrical output dramatically decreased. The decrement in voltage was 83% (from 82.5 V to 14.1 V), and the current decreased by 90% (from 4.21 μA to 0.42 μA). After natural drying, each electrical output recovered to its initial value, and this trend was observed in the remaining two repeated experiments. Considering these results, the proposed AFT-OGH can stably generate electrical output under various conditions, such as humidity and different input frequencies.

**Figure 4:**
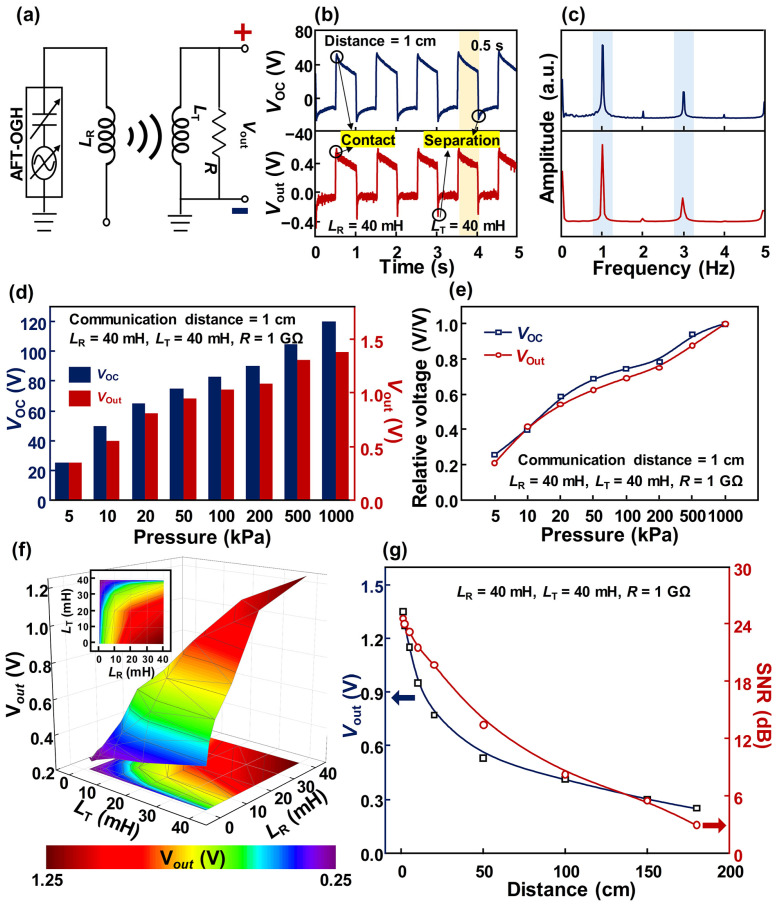
Demonstration of self-powered and real-time wireless communication: (**a**) Circuit diagram for self-powered and real-time wireless communication. The measured electrical outputs generated from the AFT-OGH and transmitting part in terms of (**b**) time and (**c**) frequency domains. *V*_OC_s and transmitted electrical outputs according to the (**d**) applied pressure and (**e**) relative values to confirm the trend line. (**f**) Transmitted electrical outputs generated from the AFT-OGH according to the inductances, and inset figure projecting the electrical outputs. (**g**) Transmitted electrical outputs and SNR values according to the communication distance.

Figure 4 demonstrates the implementation of self-powered, real-time wireless communication using two inductors. In a previous study, triboelectric-induced magnetic fields were utilized for self-powered, real-time wireless communication [50]. In Figure 4a, the circuit diagram was proposed for self-powered, real-time wireless communication using the AFT-OGH. Because the AFT-OGH generated AC electricity, this AC electricity can create a magnetic field after passing through the receiving inductor (*L*_R_). This triboelectric-induced magnetic field then induces electricity in the transmitting inductor (*L*_T_). In Figure 4b,c, the triboelectricity and the induced electricity were analyzed in both the time and frequency domains. The waveforms of the triboelectricity and induced electricity displayed high similarity in shape. Additionally, the time duration from the highest to the lowest value of both signals was similar, at 0.5 s. In the frequency domain, the first and second dominant frequencies of each signal were the same. Moreover, in Figure 4d, the *V*_OC_ generated from the AFT-OGH and output voltage measured at the transmitting part were investigated by varying the input pressure, with relative voltage values shown in Figure 4e. Both electrical outputs exhibited a similar increasing trend as input pressure increased. Considering these results, the induced electricity originated from the triboelectricity due to their high similarity. In Figure 4f, the effect of inductance on the transmitted voltage was investigated with a communication distance of 1 cm. Due to mutual inductance between *L*_T_ and *L*_R_, the measured output voltage at the transmitting part increased proportionally with the inductance. The inset figure projects a 2D graph of Figure 4f, confirming that the highest output voltage was measured at an inductance of 40 mH for both *L*_T_ and *L*_R_. This result coincides with the previous study [50]. In Figure 4g, the effect of the communication distance on the output voltage was confirmed, and the signal-to-noise ratio (SNR) was calculated simultaneously. The proposed method showed a communication distance of 200 cm, as shown in Figure S1. However, errors can easily occur because the difference between the noise and the generated signal is not large. Therefore, the proposed self-powered, real-time wireless communication system achieves wireless communication over a distance of 180 cm by minimizing communication errors. These results demonstrate the capability of the proposed AFT-OGH to function as a contact detection sensor, as well as its suitability for integration into self-powered, real-time wireless communication systems.

**Figure 5:**
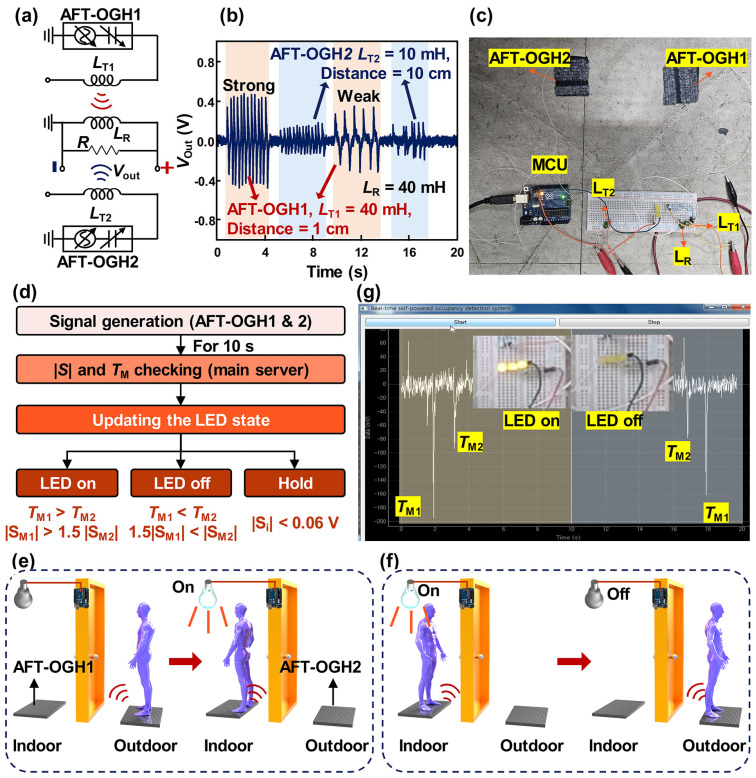
Self-powered occupancy detection system: (**a**) Circuit diagram of T-MISO system and (**b**) its electrical output. Optical image for (**c**) AS-MODS and (**d**) its algorithm for occupancy detection. Illustrations for (**e**) entering and (**f**) exiting to explain the proposed algorithm. (**g**) Electrical output measured by AS-MODS.

Figure 5 demonstrates the self-powered, real-time wireless communication-based occupancy detection system. In Figure 5a, a circuit diagram of a triboelectricity-driven multiple-input–single-output (T-MISO) system using two AFT-OGHs and inductors is shown. The effects of the inductance and distance on the output voltage at the transmitting part were previously investigated in Figure 4f,g. Based on these results, a 40 mH inductor (*L*_T1_) was utilized at the transmitting part connected to the AFT-OGH1, with a communication distance of 1 cm. Another transmitting part utilized a 10 mH inductor (*L*_T2_) with a communication distance of 10 cm. When contact events occurred in the sequence of AFT-OGH1 and AFT-OGH2, each contact event was successfully detected by the single receiving part, as shown in Figure 5b, demonstrating the successful operation of the T-MISO system. Adjusting only one parameter, such as communication distance or inductance, may be sufficient; however, considering the characteristic of the AFT-OGH that the amplitude of electrical output depends on the strength of mechanical movement, modulation of both parameters provides a more accurate distinction.

After demonstrating the T-MISO system, the AFT-OGH-driven self-powered T-MISO occupancy detection system (AS-MODS) was implemented, as shown in Figure 5c. This system was composed of the T-MISO system (two AFT-OGHs, three inductors, and load resistance), a main server, and three LEDs. To detect the presence of a user in the space, a suitable algorithm was designed, as shown in Figure 5d. The *S*_Mi_ and *T*_Mi_ are the *i*-th highest value of the electrical signal and time when the *i*-th highest value of the electrical signal occurs, where *i* is a natural number. The demonstration video is provided in Supporting Video S1. Additionally, the illustration of the working process of the proposed AS-MODS is shown in Figure 5e,f. By comparing *T*_M1_ and *T*_M2_ with *S*_M1_ and *S*_M2_ simultaneously, the movement of the user can be determined. The detailed logic flow for detecting the movement of the user is explained. Firstly, two AFT-OGHs are located on each side of the door. When a user begins to approach the door, the AFT-OGH2 generates electricity, and the receiving part transmits this signal. Then, the user inevitably steps on the AFT-OGH1 when opening the door and proceeding inside, generating a second signal, as shown in the yellow region in Figure 5g. The AFT-OGH1 was connected with the 40 mH inductor, and the communication distance was 1 cm; *S*_M1_ and *T*_M1_ were assigned to the signal generated from the AFT-OGH1, and *S*_M2_ and *T*_M2_ were allocated to the signal generated from the AFT-OGH2. Hence, when the AS-MODS confirms corresponding signals, the LEDs are turned on. In contrast, when the user exits from the inside, *T*_M2_ occurs before *T*_M1_. The LEDs are turned off when the AS-MODS detects this signal, as shown in the blue region in Figure 5g. When the user remains in position, only noise signals occur, which do not differ significantly in amplitude. Considering this, the AS-MODS maintains the LEDs’ state when the |*S*_Mi_| is lower than 0.06 V. The demonstration video is provided in Supporting Video S2. Considering these results, the proposed AS-MODS is expected to be utilized as a smart energy management system in the near future, because this system shows great potential to contribute to the decrease in energy consumption.

## 4. Conclusions

In summary, an all-fiber-based triboelectric nanogenerator (AFT) was fabricated using conductive PET fabric (CPF). By conducting a treatment process composed of O_2_ plasma treatment and GO/TA solution coating before sputtering, and hexane and 1-octadecanethiol coating (OGH) on the CPF, the AFT-OGH was fabricated, and its stability was successfully improved in terms of sheet resistance and bending stress. Then, the electrical output generated from the AFT-OGH and the effect of each CPF on the electrical output were investigated. The AFT-OGH generated a high electrical power density of 0.35 W/m^2^. The AFT-OGH can generate stable electrical output when external conditions, such as input frequency and relative humidity, are changed. Also, the AFT-OGH showed no significant degradation during a 10 h durability test, and it maintained its electrical performance even after water droplets were applied and dried. Self-powered and real-time wireless communication was successfully demonstrated using the proposed AFT-OGH, and wireless communication with a communication distance of 180 cm was achieved without an external power supply. Based on these results, the triboelectricity-driven multiple-input–single-output (T-MISO) system was demonstrated for self-powered and real-time wireless communication. The proposed T-MISO system successfully detected each signal generated from the two AFT-OGHs, and an AFT-OGH-driven self-powered T-MISO occupancy detection system (AS-MODS) was implemented. With the unique algorithm, the proposed AS-MODS successfully detected the existence of users in a specific space and controlled the LEDs depending on user occupancy, indicating an effective energy management strategy. Considering these results, the proposed AS-MODS is expected to be utilized as a smart energy management system in the near future, due to its excellent potential to manage energy consumption.

## Figures and Tables

**Table 1:** Definitions of abbreviations.

Abbreviation	Definition	Abbreviation	Definition
CPF	Conductive PET fabric	CPF-NT	CPF without any treatment
OG	Surface treatment process composed of O_2_ plasma treatment and GO/TA coating	CPF-G	CPF with only GO/TA coating
OGH	OG with hexane and 1-octadecanethiol coating	AFT	All-fiber-based TENG
		AFT-OGH	AFT using CPF-OGH
CPF-OGH	CPF conducting OGH	AFT-OG	AFT using CPF-OG
CPF-OG	CPF conducting OG	AFT-NT	AFT using CPF-NT

## Data Availability

Data are contained within the article.

## Supplementary Materials

The following supporting information can be downloaded at: https://www.mdpi.com/article/10.3390/polym17010034/s1, Table S1: Optimal load resistance, output voltage, output current, and power density of the AFT-OGH, AFT-OG, and AFT-NT. Figure S1: The output voltage measured from the transmitted signal of self-powered, real-time wireless communication with a distance of 200 cm. Video S1: Demonstration of the AS-MODS showing the algorithm to detect the user. Video S2: Demonstration of occupancy detection sensor using the AS-MODS.
