# Supplementary material for: Self-Powered Triboelectricity-Driven Multiple-Input–Single-Output Occupancy Detection System Using a Triboelectric Nanogenerator for Energy Management
Source: Polymers (Basel). 2024 Dec 26;17(1):34. doi: 10.3390/polym17010034 (PMC11722702; doi:10.3390/polym17010034)

Self-Powered Triboelectricity-Driven Multiple-Input–Single-Output Occupancy Detection System Using a Triboelectric Nanogenerator for Energy Management

Jonghyeon Yun[1] and Daewon Kim[2]*

[1]Department of Electronics and Information Convergence Engineering, Institute for Wearable Convergence Electronics, Kyung Hee University, 1732 Deogyeong-daero, Giheung-gu, Yongin, 17104 Republic of Korea

[2]Department of Electronic Engineering, Institute for Wearable Convergence Electronics, Kyung Hee University, 1732 Deogyeong-daero, Giheung-gu, Yongin, 17104 Republic of Korea

*Corresponding author: Daewon Kim (E-mail: daewon@khu.ac.kr)

Table of contents

1. **The Table for optimal load resistance, output voltage, output current, and power density of the AFT-OGH, AFT-OG, AFT-NT**
2. **The output voltage measured from the transmitted signal by the self-powered, real time wireless communication with the distance of 200 cm.**
3. **The demonstration video for the AS-MODS showing the algorithm to detect the user.**
4. **The demonstration video of occupancy detection sensor using the AS-MODS.**

1. The Table for optimal load resistance, output voltage, output current, and power density of the AFT-OGH, AFT-OG, AFT-NT. (Table S1)

	**AFT-OGH (4 cm^2)**	**AFT-OG (4 cm^2)**	**AFT-NT (4 cm^2)**
Load resistance (Ω)	20 M	20 M	50 M
Output voltage (V)	53	47	43
Output current (μA)	0.91	0.74	0.46
Power density (W/m^2)	0.35	0.28	0.09

2. The output voltage measured from the transmitted signal by the self-powered, real time wireless communication with the distance of 200 cm (Figure S1).

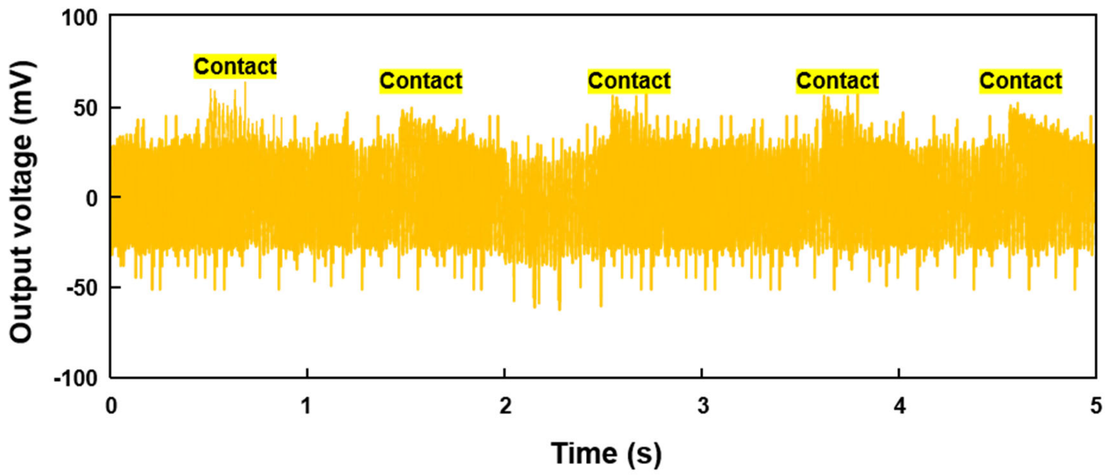

Supplement: Supplementary file 1 [file polymers-17-00034-s001.zip › polymers-3372654-supplementary.pdf]
